# Evaluation of Anemia in the Older Population in a Tertiary Care Hospital

**DOI:** 10.7759/cureus.97109

**Published:** 2025-11-17

**Authors:** Ashish Malla, Abhishek P Dash, Sourabh Pradhan, Ganguri Supraja, Samir Sahu, Sobhitendu Kabi

**Affiliations:** 1 General Medicine, Institute of Medical Sciences (IMS) &amp; Siksha ‘O’ Anusandhan University Medical (SUM) Hospital, Siksha ‘O’ Anusandhan, Deemed to be University, Bhubaneswar, IND

**Keywords:** anaemia of chronic disease, elderly patients, haemoglobin, ida, mortality

## Abstract

Background

The prevalence and severity of anemia among older people are significant health concerns. Hence, the initial medical service provider for older people with any symptoms or signs admitted under any department should be aware of the blood hemoglobin status and if anemia is present and its severity at the very outset, rather than later. This can help treat the presenting problem, along with the workup and management of anemia at the earliest. Hence, we thought of assessing the severity of anemia in the older age group in the eastern coastal belt of India.

Methods

A prospective observational study was conducted among 197 individuals of more than 64 years of age who were hospitalized over a period of 18 months. Evaluation of anemia is done by complete blood count, comment on peripheral smear, liver function test, renal function test, iron profile, stool occult blood test, and measurement of vitamin B12 and folate levels. Invasive investigations like endoscopy, colonoscopy, and bone marrow aspiration were done in indicated patients.

Results

During the study period, 197 patients with anemia were evaluated, out of which 121 were male and 76 were female. In our study, iron deficiency anemia (IDA) was the most common cause in 31.9% patients, followed by anemia of chronic disease in 22.8% and hematological disorders in 11.2%.

Conclusion

From this study, IDA is the most common cause of anemia among elderly patients. Evaluation and management of anemia at the earliest can reduce the mortality and morbidity among elderly patients.

## Introduction

Anemia is the most common hematological abnormality in the older population [1]. Older adults represent a key demographic that is growing rapidly, particularly with the increasing occurrence of anemia in aging individuals [2].

Globally, populations are aging, with both the number and percentage of older adults rising steadily. In India, the elderly population has grown significantly, increasing over four times from 24.7 million in 1961 to 103.8 million in 2011. During this period, the proportion of elderly individuals also rose from 5.6% to 8.6% [3].

According to the World Health Organization (WHO), anemia is diagnosed when hemoglobin (Hb) levels fall below 13 g/dL in men and 12 g/dL in women [4]. As people age, bone marrow function naturally declines, contributing to lower Hb levels [5]. However, if an elderly individual has Hb levels below 12 g/dL, it often indicates an underlying medical condition requiring further investigation [6].

The causes of anemia are diverse: anemia of chronic disease (ACD) is probably the most common cause in the elderly [7]. However, iron deficiency anemia (IDA) due to any underlying malignancy is also common in the elderly. The incidence and prevalence of malignancy increase with an increase in age. Anemia is prevalent in more than 60% of cancer patients, and the risk of anemia is higher with a more advanced stage of cancer [8].

Anemia in the elderly arises from multiple causes and often severely affects both daily functioning and overall well-being. It is linked to adverse consequences such as reduced physical and cognitive abilities, higher risks of falls, frailty, dementia, hospitalizations, and even mortality [9].

As anemia is very common in the elderly, the initial workup includes a complete hemogram to look for the presence of anemia, and if it is present, further workup should be done to find out the etiology. It helps in treating anemia and the underlying cause at the earliest so that the morbidity and mortality can be reduced.

The current study was undertaken to assess the severity of anemia in the elderly in the eastern coastal belt of India. Although anemia in elderly populations has been studied in various regions of India, there is a paucity of data from the eastern coastal belt, where socioeconomic, dietary, and environmental factors may differ significantly. This prospective observational study provides region-specific data on the severity and etiological distribution of anemia among hospitalized elderly patients. In addition, the study’s comprehensive diagnostic approach and evaluation of demographic associations (age, sex, and clinical presentation) contribute new insights that may inform diagnostic protocols and early management strategies in similar tertiary care settings. This study might be helpful for clinicians to have a better idea about the cause of anemia and comorbidities in those presenting with anemia at the point of admission. The etiology of anemia in the elderly in this part of India can be compared with that prevalent in other parts of the country and the world.

## Materials and methods

This prospective observational study was carried out in the Department of General Medicine at the Institute of Medical Sciences &SUM Hospital, Siksha ‘O’ Anusandhan University, Bhubaneswar, following approval from the ethical committee. Spanning 18 months (from January 1, 2020, to June 30, 2021), the research involved 197 participants, including all hospitalized patients aged over 64 years. Written informed consent was obtained from all eligible participants before their enrolment in the study.

Inclusion criteria

Patients aged more than 64 years presenting with anemia are included in our study. Furthermore, all participants' consent forms are also obtained for the study.

Exclusion criteria

The exclusion criteria include patients who do not provide consent during this study. We have excluded anemic patients who received blood transfusions within three months before data collection. Patients who developed anemia during their hospital stay due to acute illness, surgery, or iatrogenic causes were excluded to avoid bias in prevalence estimates.

Operational definitions and diagnostic criteria

Anemia was defined according to the WHO criteria as Hb < 13 g/dL in men and <12 g/dL in women. IDA was identified by serum ferritin < 30 ng/mL, low serum iron, and elevated total iron-binding capacity (TIBC). ACD was characterized by normal or elevated ferritin levels with low serum iron and low TIBC in the presence of chronic inflammatory disease or malignancy. Megaloblastic anemia was defined as a mean corpuscular volume (MCV) > 100 fL with low serum vitamin B12 (<200 pg/mL) or folate (<3 ng/mL). Chronic kidney disease (CKD) was defined as an estimated glomerular filtration rate (eGFR) < 60 mL/min/1.73 m² for ≥3 months. Malignancy was confirmed by imaging and/or histopathology.

Data analysis

The study participants were included if they/their representatives agreed to provide consent for participation in the study. Specifically, all diagnoses were made by qualified physicians in the Department of General Medicine under the supervision of senior consultants. The diagnostic evaluation for anemia and its etiology was performed at the time of hospital admission and completed during the inpatient stay as relevant investigations became available. Details about complaints, history of present illness, personal history, and comorbidities were recorded in a proforma. Meticulous clinical examination of patients was done. Laboratory investigations like complete blood count, liver function test, renal function test, urine routine microscopy, stool occult blood test, iron profile, reticulocyte count, vitamin B12, and folic acid were done. A chest X-ray was advised for patients with a cough to rule out pulmonary tuberculosis. Upper gastrointestinal (GI) endoscopy was advised for patients who had IDA and were positive for the stool occult blood test. Colonoscopy was advised for patients who were positive for the stool occult blood test but had normal upper GI endoscopy findings. Bone marrow aspiration and biopsy were advised for patients suspected of having aplastic anemia, lymphoma, or multiple myeloma.

Statistical analysis

The generated data were recorded in Excel 2019 (Microsoft Corp., Redmond, WA, US) and exported into SPSS 21 (IBM Corp., Armonk, NY, US) for statistical evaluation. Categorical variables were presented as frequencies and percentages, with comparisons made using the Chi-squared test with p-value < 0.001 represented as statistically significant.

## Results

A total of 445 patients of more than 64 years of age were admitted to IMS & SUM Hospital. Of them, 197 subjects who consented to participate in the study were included over a period of 18 months.

The mean age of the study population was 77.1 ± 10.8 years. The majority of them (54.8%) were aged between 65 and 74 years, while 32.9% of the subjects were aged between 75 and 84 years. The male-to-female ratio was 1.6:1.

**Figure 1:**
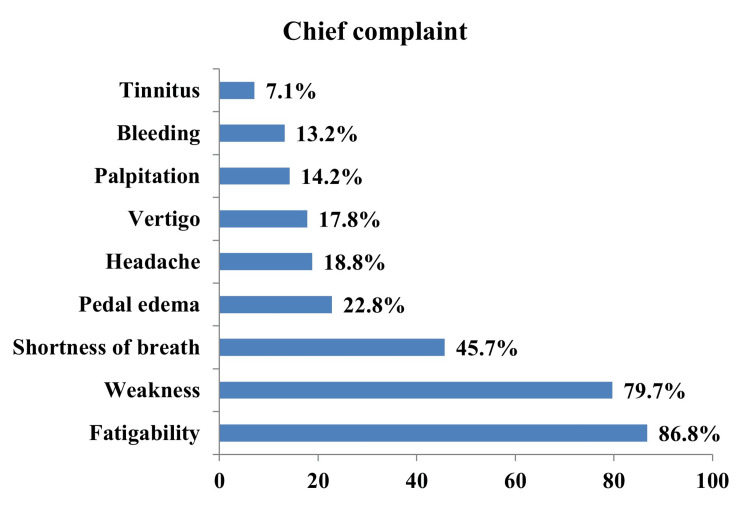
Clinical spectrum of symptoms in patients with anemia

The most common complaint was fatigability (86.8%) (Figure 1), then weakness (79.7%), breathlessness (45.7%), headache (18.8%), vertigo (17.8%), palpitation (14.2%), bleeding (13.2%), and tinnitus (7.1%). The most common physical finding was pallor (84.8%), followed by pedal edema (21.8%), and then glossitis (10.2%) (Figure 1).

In our study, approximately 47% of the patients had mild anemia, 38.6% had moderate anemia, and 14.7% had severe anemia. The mean MCV, mean corpuscular hemoglobin (MCH), MCH concentration (MCHC), red cell distribution width-coefficient of variation (RDW-CV), and hematocrit (HCT) were 89.23 ± 17.34 fL, 31.21 ± 8.4 pg, 31.56 ± 2.10%, 16.4 ± 2.67%, and 28.31 ± 7.28%, respectively. Microcytic hypochromic anemia was the most common (42.1%), followed by normocytic normochromic (27.4%), dimorphic anemia (19.2%), and macrocytic anemia (11.1%).

**Figure 2:**
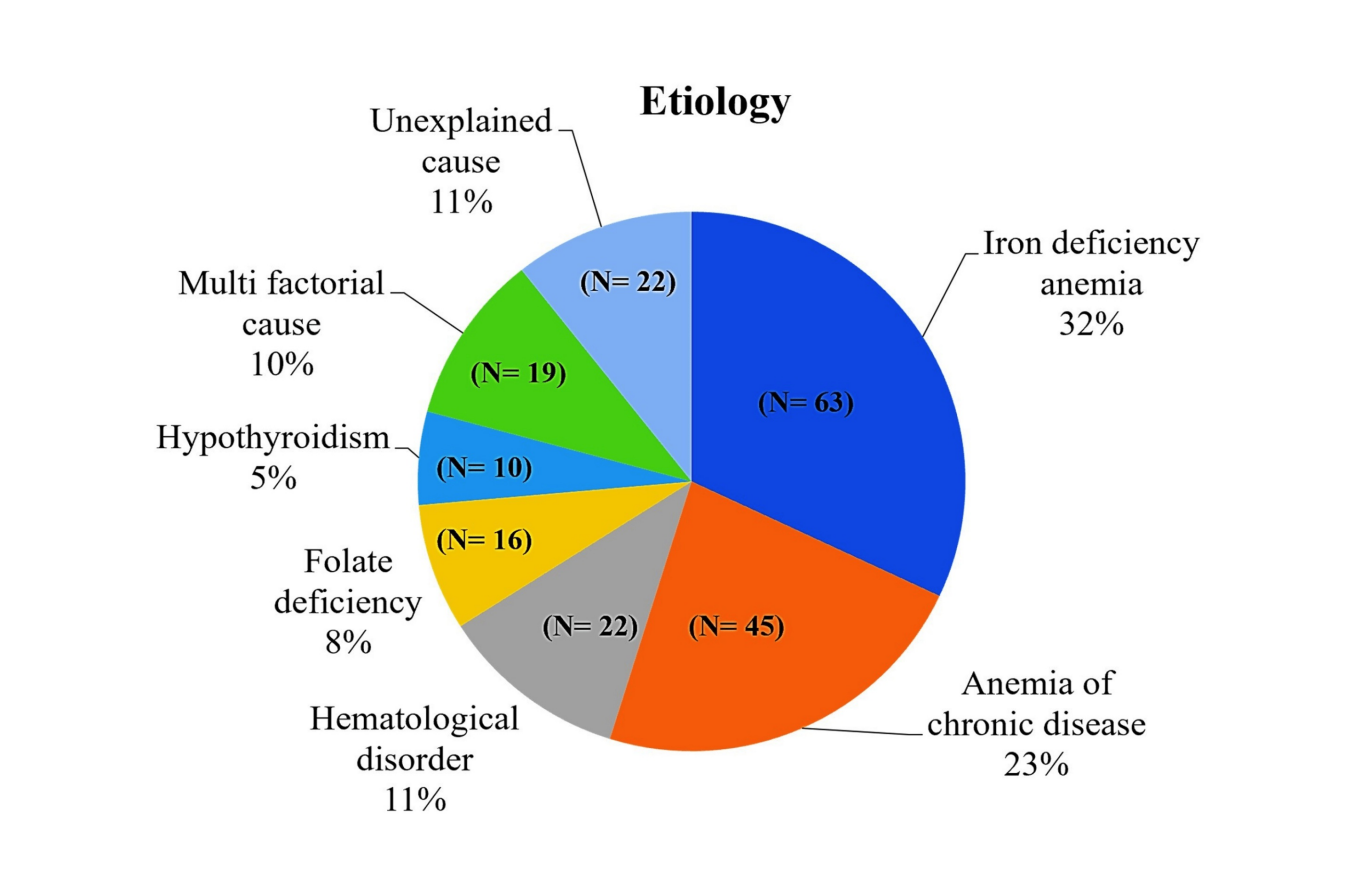
Distribution of anemia etiologies in the elderly population

In our study, as described in Figure 2, IDA was the most common in 31.9% of patients, then ACD (22.8%), and hematological disorders (11.2%). Eight percent had folate deficiency, and 5% had hypothyroidism.

Varices were the primary cause of IDA in 30.1% of individuals, followed by peptic ulcer disease (28.6%), nutritional deficiency (20.6%), and malabsorption (16.7%). Solid organ malignancy was the most common cause of ACD (37.8%), followed by CKD (22.2%). Among hematological disorders, myelodysplastic syndrome was the most common (31.8%), followed by multiple myeloma (22.7%), and chronic myeloproliferative disorders in four patients.

**Figure 3:**
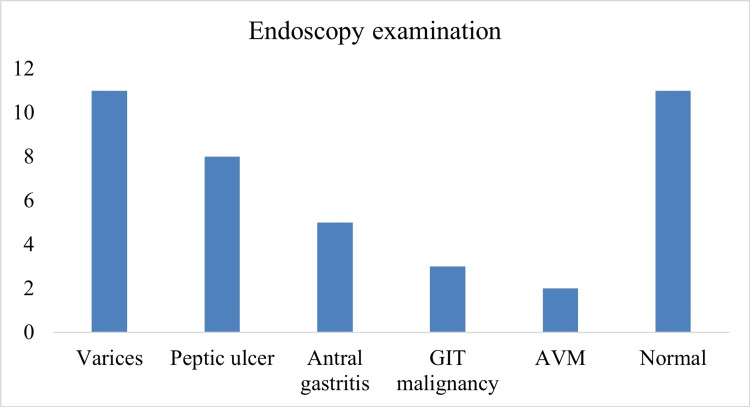
Spectrum of upper GI endoscopic lesions detected in anemia patients GI: gastrointestinal; GIT: GI tract; AVM: arteriovenous malformation

Upper GI endoscopy evaluation was advised in 63 patients who had IDA and a positive stool occult blood test, out of which endoscopy was done in 40 patients and the remaining 23 patients refused endoscopy. Endoscopic evaluation revealed varices in 27.5%, peptic ulcer in 20%, antral gastritis in 12.5%, GI malignancy in 7.5%, and arteriovenous malformation in 5% of patients (Figure 3).

**Figure 4:**
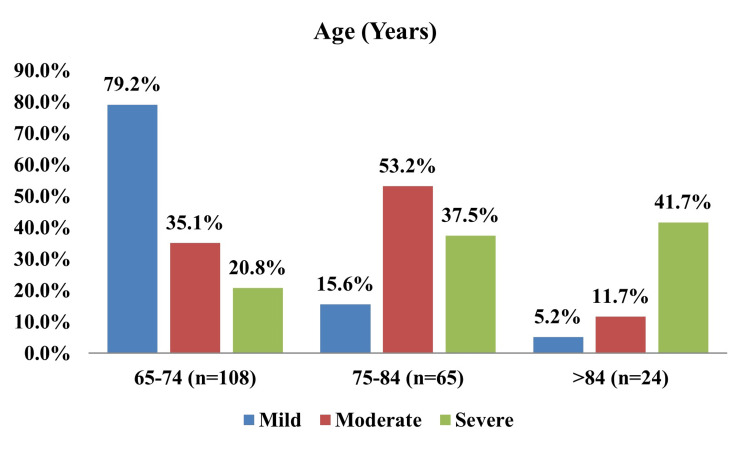
Trends in anemia severity across different age groups

Colonoscopy was carried out in those patients where endoscopy was not conclusive. Colonoscopy findings revealed bleeding colonic polyps in 20%, malignancy in 33.3%, and hemorrhoids and colitis in 6.6% patients each. In this study, higher age was significantly associated with the severity of anemia (p < 0.0001) (Figure 4 and Table 1).

**Table 1:** Severity of anemia according to age group

Age (years)	Mild	Moderate	Severe	Test statistic	Test name	p-value
65-74 (n = 108)	76 (79.2%)	27 (35.1%)	5 (20.8%)	86.84	Chi-squared test (χ²)	<0.001
75-84 (n = 65)	15 (15.6%)	41 (53.2%)	9 (37.5%)			
>84 (n = 24)	5 (5.2%)	9 (11.7%)	10 (41.7%)			
Total	96 (100%)	77 (100%)	24 (100%)			

**Figure 5:**
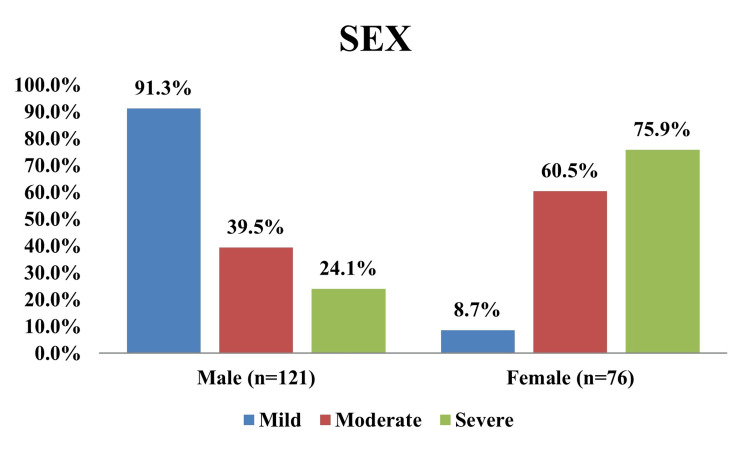
Correlation of gender with anemia severity

In this study, as shown in Figure 5 and Table 2, female sex was significantly associated with the severity of anemia (p < 0.0001).

**Table 2:** Severity of anemia according to sex

Sex (years)	Mild	Moderate	Severe	Test statistic	Test name	p-value
Male (n = 121)	84 (91.3%)	30 (39.5%)	7 (24.1%)	67.13	Chi-squared test (χ²)	<0.001
Female (n = 76)	8 (8.7%)	46 (60.5%)	22 (75.9%)			
Total	92 (100%)	76 (100%)	29 (100%)			

## Discussion

The WHO estimates that the number of people aged >60 years will rise from 900 million in 2015 to two billion in 2050, which is from 12% to 22% of the global population [10]. Anemia in the elderly has been associated with various numbers of adverse outcomes, including frailty and reduced physical activity [11], decreased muscular strength causing a higher chance of falls [12], cognitive decline and dementia [13], increased risk of hospitalization with prolonged hospital stay [13], and even higher mortality risk [14]. Primarily, many of these associations remained statistically significant even after adjusting for many possible confounders, indicating that anemia by itself may have a negative impact independent of concurrent chronic conditions like CKD, congestive heart failure (CHF), and inflammatory disorders. Notably, the increased mortality rate was not limited to those with severe anemia but was also found in early individuals with mild anemia.

This study analyzed the clinical profile and causes of anemia in the elderly. In this study, the most common complaint was fatigability (86.8%), followed by weakness (79.7%) and shortness of breath (45.7%). The observations are in tune with the following studies. In the study by Bhasin and Rao, fatigue was the most common symptom, found in 74% of patients. Palpitations and anorexia were the next common symptoms, each present in 13% patients [15]. Similarly, Thyagaraja et al. reported the most common clinical presentation was easy fatigability (70%), followed by dyspnea (50%) and palpitation (40%) at the time of presentation [16].

In our study, mild anemia was present in approximately 47% of the patients, while 38.6% had moderate anemia, and 14.7% were severely anemic. In a study by Geisel et al., only 1.5% (n = 4) of their patients were severely anemic, n = 37 (13.5%) had moderate anemia, and the majority (85.1%) had mild anemia [17]. On the other hand, a study by Prakash et al. found a higher prevalence of severe anemia, with 42% (n = 21) of patients having Hb < 6 gms%, 40% (n = 20) with moderate anemia (Hb 6-9 gms%), and 18% (n = 9) with mild anemia (Hb > 9 gms%) [18]. In line with previously published results [2], our study also found that anemia was most often mild, with Hb levels more than 10 g/dL.

In this study, microcytic hypochromic anemia was the most common (34.7%), followed by dimorphic anemia (30.6%), normocytic normochromic (19.4%), and macrocytic anemia (15.3%). In the study by Bhasin and Rao, microcytic anemia was the most common in 30% of patients. In the study by Thyagaraja et al., the most common type of anemia was microcytic hypochromic anemia (38% of patients) [16]. In India, chronic blood loss and dietary iron deficiency are also causes of microcytic hypochromic anemia.

In our study, IDA was the most common in 31.9% of patients, followed by ACD (22.8%) and hematological disorders (11.2%). Upper GI ulcers were the primary cause of IDA in 28.6%, followed by nutritional deficiency (20.6%) and GI malignancy (16.7%). A small number of studies have been done on the etiologic profile of anemia in patients aged from 65 to 101 years. Comparing our results with previous studies is challenging due to variations in the classification of anemia. Nevertheless, IDA remains the most common form of anemia among both community-dwelling and hospitalized geriatric patients [19]. In the elderly, iron deficiency is frequently observed, typically resulting from chronic GI tract (GIT) blood loss.

In our study, ACD (22.8%) was the second most common cause of anemia. Davenport showed that ACD is considered to be the most common cause of anemia in the world [20]. Another study done in South India by Prakash et al. also had ACD as the most common cause of anemia [18]. In the study by Thyagaraja et al., ACD was the third most common (12%) [16]. Elejalde Guerra et al. revealed that IDA is the most frequent, followed by hemorrhagic anemia and ACD [21].

In our study, endoscopic evaluation revealed varices in 27.5%, peptic ulcer in 20%, antral gastritis in 12.5%, GIT malignancy in 7.5%, and arteriovenous malformation in 5% of patients. Colonoscopy findings revealed bleeding colonic polyps in 20%, malignancy in 33.3%, and hemorrhoids and colitis in 6.6% of patients each. In the study by Bhasin and Rao, upper GI lesions were identified in 78.6% of the patients with IDA, while colonic lesions were found in 29.4% of the cases [15]. GI malignancies were detected in 6.66% of the patients, including two gastric, one esophageal, and five colonic cancers. These findings are consistent with previous studies and highlight the importance of thorough GIT evaluation.

## Conclusions

The findings from this study highlight the aetiology of anemia among hospitalized older adults in this eastern coastal region of India, with IDA identified as the most frequent etiology. ACD and hematological disorders were also significant contributors. Further, the findings also emphasize the need for early and comprehensive evaluation of blood Hb status upon admission for all older patients, regardless of their presenting symptoms. Timely diagnosis and management of anemia can significantly improve outcomes, reducing morbidity and mortality in this vulnerable population. Further research is warranted to explore the specific causes of IDA in this demographic and to develop targeted intervention strategies.
